# Ultrasound-guided percutaneous procedures in pancreatic diseases: new techniques and applications

**DOI:** 10.1186/s41747-018-0081-2

**Published:** 2019-01-22

**Authors:** Mirko D’Onofrio, Alessandro Beleù, Riccardo De Robertis

**Affiliations:** 1 Department of Radiology, G.B. Rossi Hospital – University of Verona, Piazzale L.A. Scuro 10, 37134 Verona, Italy; 2 PhD Programme in Inflammation, Immunity and Cancer, University of Verona, Piazzale L.A. Scuro 10, 37134 Verona, Italy

**Keywords:** Cryosurgery, Electroporation, Microwaves, Pancreatic diseases, Radiology (interventional), Ultrasonography

## Abstract

Ultrasound (US) is not only an important diagnostic tool for the evaluation of the pancreas, but is also a fundamental imaging technique to guide percutaneous interventions for several pancreatic diseases (fluid aspiration and drainage; invasive diagnosis by means fine-needle aspiration and core-needle biopsy; tumour ablation by radiofrequency, microwaves, irreversible electroporation, cryoablation, and high-intensity focused US). Technical improvements, such as contrast media and fusion imaging, have recently increased precision and safety and reduced procedure-related complications. New treatment US techniques for the ablation of pancreatic tumours, such as contrast-enhanced US and multimodality fusion imaging, have been recently developed and have elicited a growing interest worldwide. The purpose of this article was to review the most up-to-date role of US in percutaneous procedures for pancreatic diseases.

## Key points

- Ultrasound is a fundamental imaging guidance in percutaneous intervention for pancreatic diseases.
- Technical improvements have increased precision and safety of percutaneous ultrasound-guided interventions.
- Percutaneous ultrasound-guided ablation of pancreatic tumours has been recently developed.

## Background

Ultrasound (US) has a central role in the evaluation of pancreatic diseases, especially in European and Asiatic Countries. Over the last decades, there have been continuous improvements in both US technology and specialists’ expertise, which expanded the capabilities of US during percutaneous intervention in several pancreatic diseases. Transabdominal US is faster and cheaper than computed tomography (CT), magnetic resonance imaging (MRI), and endoscopic US (EUS). However, US is strongly dependent on operator expertise, in particular when used as a guidance for interventional procedures for pancreatic diseases. In this case, transabdominal US is particularly helpful for minimally invasive procedures with percutaneous approach, as it guarantees a real-time imaging that allows to precisely evaluate each step of the procedure.

The most commonly performed percutaneous US-guided procedures on the pancreas are fluid drainage, especially after surgery or acute pancreatitis, and invasive diagnostic of pancreatic masses. Recently, several percutaneous ablative treatments, which have a proven therapeutic role for hepatic and renal malignancies, have been applied to pancreatic malignancies. Differently from fluoroscopy and CT, when percutaneous intervention is performed under US guidance, it is possible to compress the patient’s abdominal wall with the US probe in order to displace intraperitoneal organs and bowels, thus reducing both the length of the path from the skin to the target and the superimposition of air, which are essential to minimise complications.

The purpose of this article was to review the most up-to-date role of US in percutaneous procedures for pancreatic diseases, also focusing on new techniques and applications.

## Fluid aspiration and drainage

Aspiration and drainage of peripancreatic fluid collections is frequently performed percutaneously under US guidance. Fluid collections are common after pancreatic surgery. They may represent different pathological entities such as exudate, bile, blood, or infection. Surgical drainage tubes are usually present when fluid collections are seen at post-operative imaging, making easier their characterisation; when drainage tubes are absent, percutaneous US-guided diagnostic aspiration should be performed to guide further management. Generally speaking, almost every symptomatic post-operative fluid collection should undergo percutaneous drainage; percutaneous drainage is mandatory when signs of superimposed infection are present. According to the latest recommendations proposed by the International Study Group for Pancreatic Fistula, percutaneous drainage of post-operative collections related to a pancreatic fistula should be performed only in patients with a grade B post-operative pancreatic fistula [1]. Peri- or intrapancreatic fluid collections are typically associated with acute pancreatitis and almost always resolve without any treatment [2].

According to the revised Atlanta classification [3], the severity or stage of acute pancreatitis drive the type of treatment that the patient needs. About 25% pseudocysts associated with interstitial acute pancreatitis become symptomatic or infected and necessitate drainage [4]. Percutaneous US-guided drainage has proved to be an effective alternative to surgery in patients with acute necrotising pancreatitis; nevertheless, the approaches to sterile and infected necrotic collections are different. Necrotic collections without signs of infection at CT should be considered as sterile until otherwise proven, and percutaneous drainage should be avoided, as this procedure has the potential of infection by means of colonisation of the drainage catheter [4]. Nevertheless, patients without radiologic evidence of infection, who do not do well clinically or present clinical instability, may benefit from US-guided aspiration to rule out infected necrosis. When infected necrosis is present, large-sized, or multiple, percutaneous drainage catheters should be placed into the collection as a bridge or as an alternative to surgical debridement.

## Invasive diagnosis of pancreatic lesions

Although all imaging techniques can be used to manage pancreatic fine-needle aspiration (FNA) and core-needle biopsy (CNB) of pancreatic lesions, US is certainly one of the most used. The endoscopic approach has been increasingly used worldwide for tissue sampling in pancreatic diseases. However, EUS guidance is not available in all centres, it is expensive and time-consuming, and requires at least deep sedation of the patient.

The most recent guidelines of the European Federation of Society for Ultrasound in Medicine and Biology (EFSUMB) on diagnostic US-guided interventional procedures [5] provided the following indications to invasive diagnosis of pancreatic lesions: characterisation of a solid unresectable pancreatic mass; differential diagnosis between neoplasm and focal inflammatory conditions; suspicion of an uncommon entity (i.e. metastases, lymphoma), even if resectable, which could be treated non-operatively; Ki-67 quantification for the prognosis of neuroendocrine neoplasms; cystic lesions that are undefined or suspicious for malignancy after MR imaging evaluation.

**Fig. 1:**
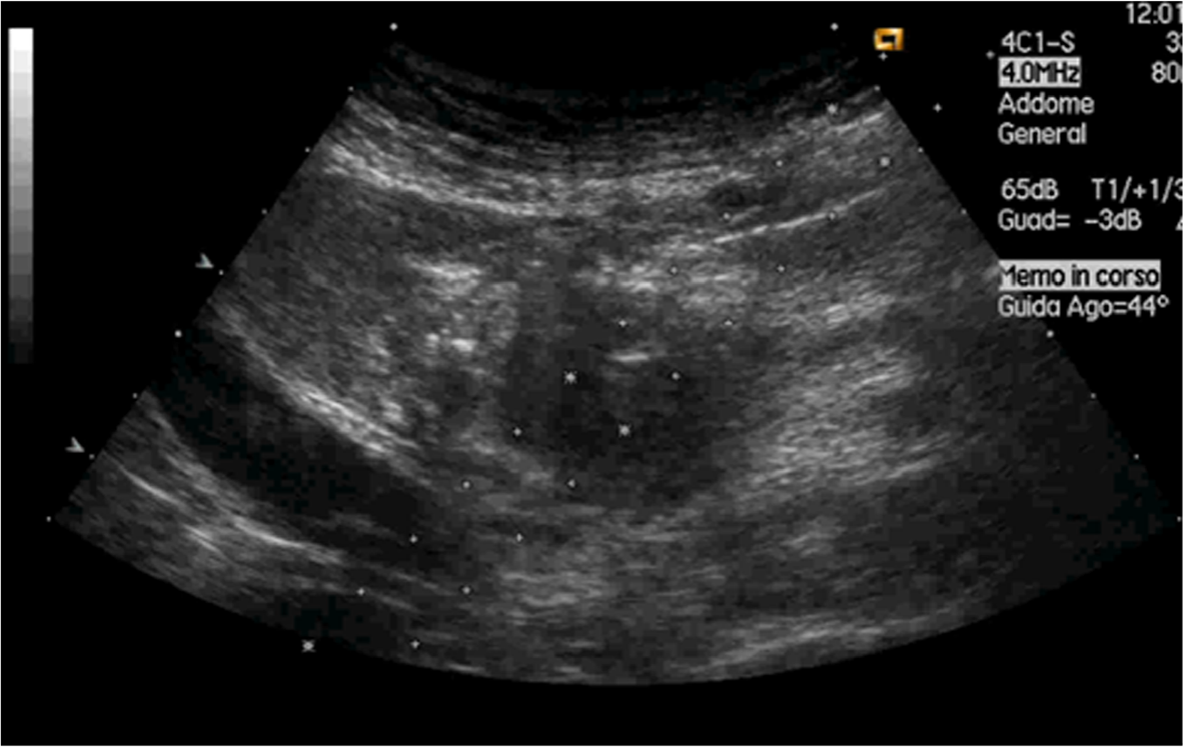
Ultrasound-guided pancreatic lesion biopsy. The path of the needle can be precisely visualised during the planning phase (*dotted line*). The tip of the needle can be exactly visualised during its insertion and stopped when in the target lesion (*hyperechoic spot*)

The same guidelines recommended that unresectable, locally advanced pancreatic masses should be evaluated for percutaneous US-guided biopsy first (Fig. 1), and if percutaneous approach is not feasible, then EUS should be considered; moreover, cystic lesions that require pathological diagnosis should be always sampled through an endoscopic approach. Contraindications to the procedure include uncooperative patients and non-correctable bleeding disorders.

Fine-needle aspiration needles range from 23G to 20G in calibre [6]. Menghini-modified needles work with an aspiration modality, while Chiba needles collect cells through capillarity; several studies have reported the superiority of aspiration needles, in particular for lesions with a low cellular density, as pancreatic ductal adenocarcinoma [7, 8]. Previous studies reported high sensitivity and accuracy values of percutaneous US-FNA for the diagnosis of pancreatic masses, even above 98% [9–11], which are comparable to those of EUS-FNA [12]. Moreover, percutaneous FNA have similar and relatively low complication rate compared with EUS-FNA, ranging between 0 and 5%, and almost always limited to post-procedural pain or mild abdominal effusion [10–14].

When a complete tissue analysis is needed for a correct histological diagnosis and for further pathological analyses, as Ki-67 quantification in neuroendocrine neoplasms, FNA is not adequate, as it only provides a cytological specimen with few histologic structures. Moreover, when FNA is performed without the immediate evaluation of the specimen by a cytopathologist, the procedure must be repeated in a different session if the sample results inadequate for a final pathological diagnosis. Core-needle biopsy (CNB) overcomes all these limitations, because it provides preserved tissue structures for histologic analysis and molecular characterisation. Coaxial cutting needles are commonly used for CNB. No significant differences in complication rates have been reported between different calibres of the CNB needle, which normally ranges between 14 to 20 G [6, 15]. Several studies have reported very high sensitivity, specificity and accuracy values for CNB of pancreatic masses, with a diagnostic rate that ranges between 92 and 96% [6]. Percutaneous CNB has a higher risk of complications [6] compared with US-FNA [10]. Therefore, percutaneous US-FNA, especially when performed in the presence of an experienced cytopathologist, has sensitivity and accuracy values comparable to those of EUS-FNA and US-CNB, but it is cheaper and with less complications.

## Tumour ablation

**Fig. 2:**
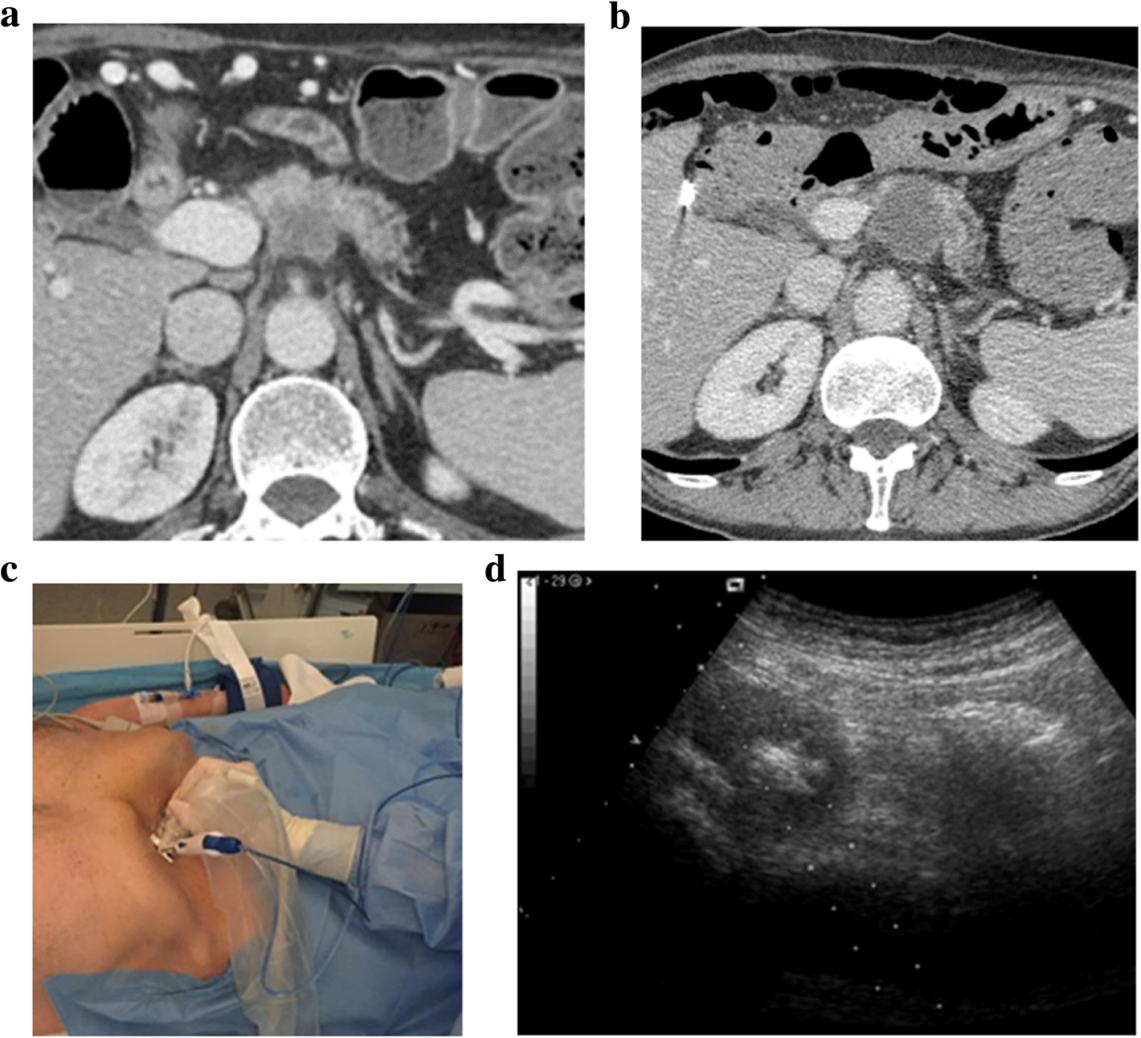
Computed tomography of an unresectable pancreatic ductal adenocarcinoma before (**a**) and after radiofrequency ablation (**b**). Patient presented with a locally advanced pancreatic ductal adenocarcinoma (40 × 35 mm) involving the celiac trunk. After twelve cycles of FOLFIRINOX chemotherapy, RFA of the lesion was performed. After the procedure (**b**), a homogeneous well-demarcated hypodense necrotic area confirmed the success of the procedure. No complications were reported. **c** Radiofrequency ablation of a ductal adenocarcinoma (patient setting). The procedure is performed in absolute sterility, in a surgery room with anaesthesia support. The ablation needle is mounted on a specific support for the probe. The procedure is performed by a single skilled operator. **d** Radiofrequency ablation of a ductal adenocarcinoma under ultrasound guidance. Gas bubbles generated during the procedure spreads centrifugally from the tip of the needle, permitting to monitor the margins of the ablated area in relation to the tumour borders

Radical resection is the only treatment capable of improving long-term survival in patients with pancreatic cancer. Surgical resection is possible only in 20–30% of patients with pancreatic cancer and the 5-year survival rate is still very low, even in combination with chemotherapy and radiotherapy [16]. Tumour ablation was first proposed under intraoperative US to debulk tumours that were found to be unresectable during surgery, basing on previous effective experiences in other organs as the liver or the kidney [17]. Afterwards, given the efficacy in terms of mass shrinkage, pain relief, CA 19.9 reduction, and survival, this procedure has been introduced as a part of the multidisciplinary approach to patients with pancreatic cancer in high-volume centres [18, 19]. As a consequence, there was the need for minimally invasive (i.e. laparoscopic, percutaneous, and endoscopic) approaches (Fig. 2) to avoid unnecessary laparotomies.

There are many ablative techniques for pancreatic cancer, which can be divided in three groups: invasive, thermal techniques, such as radiofrequency ablation (RFA), microwave ablation (MWA), laser ablation, and cryoablation; invasive, non-thermal techniques, such as ethanol injection and irreversible electroporation (IRE); and non-invasive ablative techniques, as high-intensity focused US (HIFU). Among these techniques, RFA and IRE are the most used for the ablation of pancreatic cancer; HIFU is an emerging alternative.

*Radiofrequency ablation* induces coagulative necrosis within the tumour mass through the production of high temperatures, induced by the application of high-frequency alternating current. While EUS is safer for lesions in the pancreatic head, the percutaneous approach can be adopted for lesions located in the body of the pancreas [20]. The necrotic area produced by RFA depends on the type of the needle-electrode. Moreover, technical parameters, as power, influence the temperature and the volume of necrosis. In the pancreas, the use of very high temperature (above 100 °C) is related to a high risk of complications without significant advantages, so several studies have shown that a temperature of about 90 °C is sufficient for a successful procedure, with lower risk of complications [17, 21, 22]. Previous studies reported that carbohydrate antigen (CA) 19.9 blood levels are reduced after RFA of unresectable pancreatic cancer, thus indirectly suggesting effective cytolysis of the tumour after ablation [23]. It has been proven that RFA can provide a reduction in back pain and analgesia requirement in inoperable patients [24]. Overall survival is longer in patients treated with RFA instead of classical supportive care, especially when combined with chemotherapy, reaching up to 33 months in unresectable pancreatic cancer [25, 26].

Despite the successful results, there are still few studies regarding percutaneous RFA of pancreatic lesions. However, all authors agreed on the safety and effectiveness of the procedure, not only for ductal adenocarcinoma [25] but also in neuroendocrine tumours [27, 28] and pancreatic metastases [29]. Owing to the above mentioned results, US-guided RFA has been introduced in the multidisciplinary approach to pancreatic cancer in high-volume centres. Nevertheless, randomised clinical trials on larger samples are needed in the future to validate this procedure.

*Microwave ablation* is based on tissue heating by mechanical agitation of water molecules induced by microwaves, which ultimately causes coagulative necrosis [30]. Microwaves can spread throughout tissues independently from their electric impedance: this allows to produce faster and larger ablation areas than RFA, thus requiring less applications to obtain complete tumour necrosis [30]. Although literature reports on percutaneous US-guided MWA of pancreatic lesions are few, this technique appears to be safe and promising for the treatment of unresectable pancreatic tumours. Carrafiello et al. [31] reported that this procedure was feasible in all patients of their series, with only one procedure-related complication. Ierardi et al. [32] reported improvement in quality of life after US-guided percutaneous MWA in five patients with pancreatic cancer.

*Irreversible electroporation* is the newest and most promising invasive technique for pancreatic cancer ablation. IRE is based on the application of short high-voltage electric pulses, in order to produce multiple micropores on cell membranes causing an irreversible permeabilisation, which leads to disruption of cellular homeostasis, activating apoptotic pathways in tumour cells [33]. The main advantage of IRE compared with other ablative techniques is the ability to preserve the extracellular matrix, thus allowing ablation adjacent to critical structures as nerves, vessels and biliary ducts; IRE is therefore the safest ablative approach for tumours encasing major peripancreatic vessels [33]. Irreversible electroporation has been proposed for palliation of unresectable tumours of the pancreas, as a bridge therapy before surgery, and also as a technique for intraoperative “margin augmentation”, in order to reach R0 resection in technically unresectable pancreatic tumours [34]. Open, laparoscopic and percutaneous approaches have been evaluated for IRE. In most cases, percutaneous IRE was performed under CT guidance, with encouraging results in terms of feasibility, safety and effectiveness [35, 36]. Preliminary studies [37, 38] reported successful percutaneous US-guided IRE of pancreatic cancer, without significant procedure-related complications. Månsson et al. [39] reported a median survival of seven months after percutaneous US-guided IRE of pancreatic cancer; the median time from IRE was 6.1 months to local progression and 2.7 months to observation of metastases. With larger studies, data on safety and overall survival after percutaneous US-guided IRE could be obtained to confirm its long-term efficacy within a multidisciplinary approach to unresectable pancreatic cancer.

*Cryoablation* is increasingly used for the ablation of unresectable pancreatic cancer. This technique produces a rapid freezing of the lesion down to temperatures between −80° and −160 °C by using a cryoprobe. The biological mechanisms underlying cryoablation are still not fully understood; nevertheless, it is known that this technique leads to the destruction of cell membranes and tissues’ ultrastructure, leading to delayed cell necrosis and apoptosis [40]. Ultrasound can be used to guide percutaneous cryoablation, but posterior acoustic shadowing limits visualisation, while at CT the frozen lesion appears as a hypodense “ice ball”. For these reasons, CT guidance is more frequently adopted to guide percutaneous cryoablation. Nevertheless, there have been reports on successful US-guided percutaneous cryoablation for pancreatic cancer. Niu et al. [41] reported effective pain relief after cryoablation, with a ≥ 50% reduction in pain score in 84% of patients, a 50% decrease in analgesic consumption in 69% of patients and a ≥ 20 increase in Karnofsky Performance Status score in 50% of patients. Xu et al. [42] reported complete tumour response in 20.4% patients, partial response in 38.8%, and stable disease in 30.6% after percutaneous cryosurgery associated with 125-iodine seed implantation.

*High-intensity focused ultrasound* is a non-invasive ablation technique that delivers high-intensity ultrasounds in a definite area in order to produce both thermal and mechanical damage. The target region is heated up to 60–80 °C inducing protein denaturation and tissue necrosis [43]. Both US and MR imaging can be used to guide the procedure; while MR imaging is the most commonly used technique, US has the advantage to identify and displace the bowels in order to improve the effectiveness of the procedure and reduce complications. HIFU has been proven to be an effective treatment for patient with advanced pancreatic cancer, by reducing pain in more than 80% of the cases [44–46]. Marinova et al. [47] reported that US-guided HIFU induced significant early relief of cancer-induced abdominal pain in 84% of patients, with a tumour volume reduction of 37.8  ±  18.1% after 6 weeks and 57.9  ± 25.9% six months after treatment. The median overall survival and progression-free survival were 8.3 and 6.8 months from intervention.

One of the most interesting as well as unknown side of ablative techniques is the possible role in immunogenic stimulation. It seems that tumour debris left *in situ* after ablation can induce a systemic immune response against tumour cells, affecting both eventual residual disease and metastases [48]. In particular, non-thermal techniques as well as cavitation phenomenon induced by HIFU, not providing thermal denaturation of tumour antigen, could stimulate strong cytokines production and a T-cell-mediated reaction against tumour cells [48]. Further studies are needed in this field.

## Novel US techniques for the guidance of interventional procedures

One of the greatest advances in US imaging has been the introduction of contrast media. Contrast-enhanced US (CEUS) is the only imaging technique that allows a real-time observation of the vascular network, owing to some particular features: the high-contrast and spatial resolution, the use of a blood-pool contrast medium and the real-time dynamic evaluation of tumour enhancement, filtering the background tissue signals [49].

The latest guidelines by the EFSUMB [50] provided the following recommendations for the use of CEUS prior or during US-guided pancreatic intervention: distinction between cystic neoplasms and pseudocysts; differentiation of vascular (solid) from avascular (e.g. liquid or necrotic) components of a pancreatic lesion; definition of dimensions and margins of a pancreatic lesion and its vascular components; diagnosis and follow-up of acute necrotising pancreatitis; improvement of the accuracy of percutaneous US-guided pancreatic procedures.

Previous studies reported that CEUS is superior to Doppler US for both the visualisation of intrapancreatic vessels and the relationship of pancreatic lesions with peripancreatic vessels [51]; thus, it can be helpful for percutaneous intervention in order to better evaluate the target lesion and to set up the most appropriate pathway of the biopsy needle. CEUS-guided biopsy may be helpful for pancreatic lesions that are barely visible on B-mode US, thus improving accuracy [52]. Moreover, by directing the biopsy needle towards solid, enhancing portions of the lesion, necrotic portions can be avoided, thus reducing the need for biopsy repetition [50]. As demonstrated by Mauri et al. [53] for liver lesions, intraprocedural CEUS could also be useful to instantly assess the success of pancreatic RFA, detecting incomplete ablations and then reducing the number of retreatments and overall costs.

*Multimodality fusion imaging* is a new technique that allows a real-time fusion of B-mode US imaging with previously acquired cross-sectional images, including CT, MRI, and positron emission tomography - CT (PET-CT) [54, 55]. This technique has a great potential for interventional radiology, since it associates the characteristics of two different types of imaging in a single examination, thus increasing the amount of anatomical, functional and metabolic information during US-guided procedures.

Fusion imaging is usually used to assist percutaneous procedures for challenging lesions, especially those characterised by low conspicuity on B-mode US [56]; most previous experiences on multimodal fusion imaging (MMFI) were applied to hepatic and prostatic intervention. Theoretically, the pancreas could benefit from this technique, since being a retroperitoneal organ it is poorly affected by respiratory movements that could impair real-time image fusion and synchronisation of the images. Nevertheless, there are very few literature reports on the use of MMFI techniques for US-guided percutaneous intervention in pancreatic diseases. Sofuni et al. [57] and Sumi et al. [58] reported potential usefulness of fusion imaging for the evaluation of the pancreatic tail, a well-known “blind area” for transabdominal US, and for pancreatic lesions with low conspicuity on B-mode US. Zhang et al. [59] compared the efficacy of US guidance alone and US/CT image fusion guidance in percutaneous drainage of infected walled-off necrosis following acute pancreatitis. The US/CT fusion group achieved a significantly higher imaging effective rate, and significantly lower inflammatory response indexes and severity score, than the US group; the US/CT fusion group required fewer puncture times and drainage tubes and lower rate of advanced treatment, showing higher operational success rate than the US group. Moreover, the US/CT fusion group exhibited significantly lower complications and hospital stay than the US group.

**Fig. 3:**
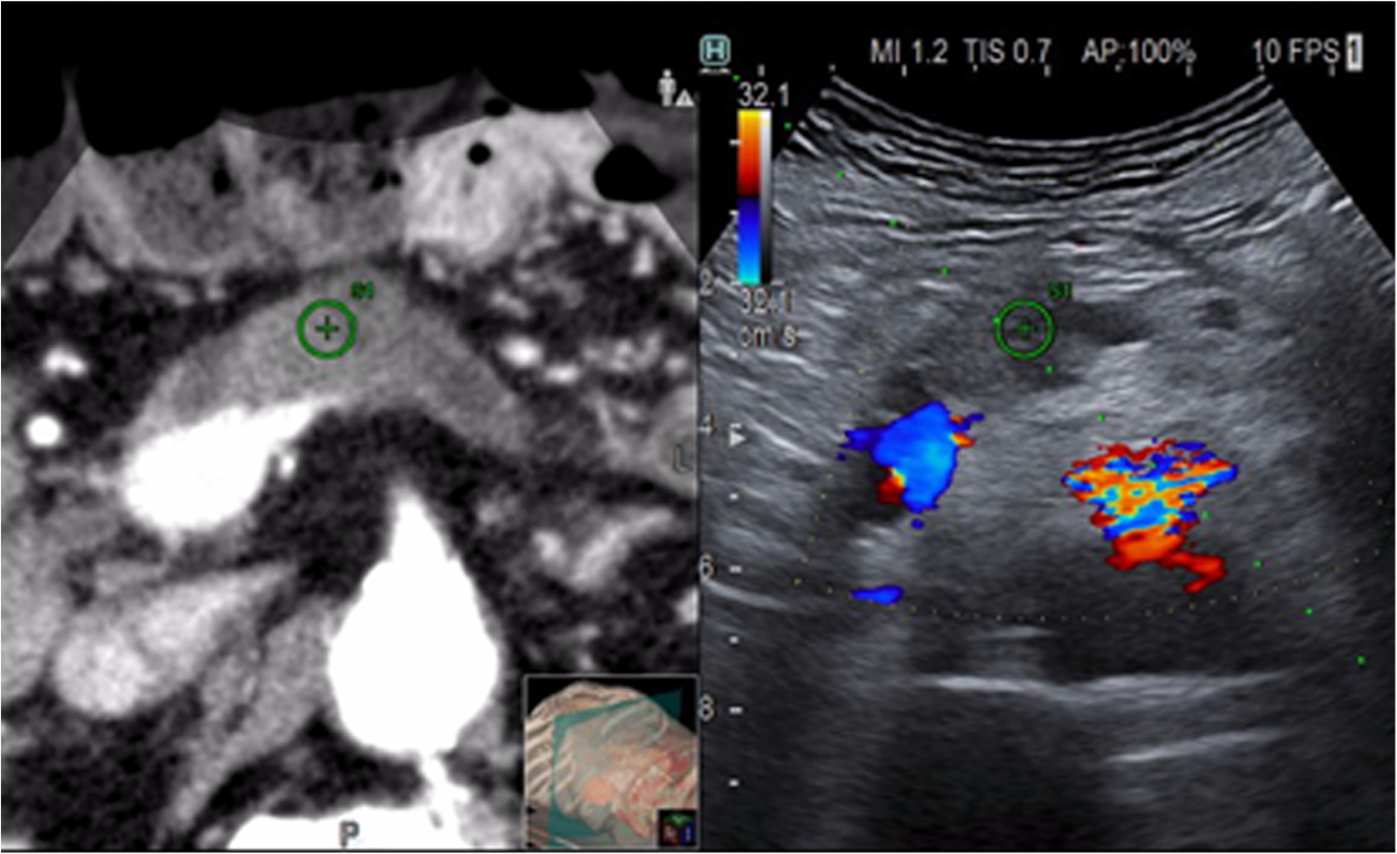
Ultrasound (US) image fused with a previously acquired computed tomography (CT). Target lesion easily is identified and marked (⊕) on both sides. Color Doppler confirms the major vessels’ relationship of the lesion well visualised on the CT on the left. Path of the needle precisely planned (*dotted line*). Interposed colon on the CT image is displaced on US by the strong compression applied by the probe

A possible limitation of fusion imaging, when applied to percutaneous pancreatic intervention, resides in the necessary compression with the US probe on the abdomen, which could create discrepancies between real-time US and previously acquired images (Fig. 3), in which no compression is applied.

## Conclusion

Ultrasound-guided percutaneous intervention for pancreatic diseases is increasingly used and is now part of clinical practice in high-volume centres all over the world. Technical advances allowed to develop and refine both diagnostic and therapeutic procedures. The use of CEUS and fusion imaging allows to increase the accuracy, safety, and feasibility of US-guided percutaneous procedures, reducing time and costs. Ablative techniques are increasingly used and may represent a therapeutic treatment within the multidisciplinary approach to pancreatic cancer.

## Abbreviations

CEUS: Contrast-enhanced ultrasound
CNB: Core-needle biopsy
CT: Computed tomography
EUS: Endoscopic ultrasound
FNA: Fine-needle aspiration
HIFU: High-intensity focused ultrasound
IRE: Irreversible electroporation
MMFI: Multimodal fusion imaging
MWA: Microwave ablation
RFA: Radiofrequency ablation
US: Ultrasound
